# Mental health and energy drink consumption among Norwegian adolescents; a cross-sectional study

**DOI:** 10.1186/s12889-025-23432-6

**Published:** 2025-06-10

**Authors:** Siri Kaldenbach, Marja Leonhardt, Tor A. Strand, Mads Holten-Andersen

**Affiliations:** 1 Department of Paediatric and Adolescent Medicine, Innlandet Hospital Trust, Lillehammer, NO Norway; 2 University of Oslo, Institute of Clinical Medicine, Oslo, NO Norway; 3 Research Centre for Substance Use Disorders and Mental Illness, Innlandet Hospital Trust, Brumunddal, NO Norway; 4 Section for Clinical Addiction Research, Oslo University Hospital, Oslo, NO Norway; 5 Research and Innovation Department, Innlandet Hospital Trust, Brumunddal, NO Norway; 6 University of Bergen, Centre for International Health, Bergen, NO Norway

**Keywords:** Energy drinks, Mental health, Public health, Adolescents

## Abstract

**Background:**

Energy drinks (ED) are marketed as boosters of mental and physical health, but few studies have looked at the mental health of adolescents who consume large amounts of ED. The current study aims to investigate the association between symptoms of depression and ED consumption among Norwegian adolescents between 2017 and 2022.

**Methods:**

Data from the Norwegian nationwide youth survey (Ungdata) with participants from lower and upper secondary schools was used. A total of 133,401 adolescents who participated between 2017 and 2022 were included. Multivariable Poisson regression models were used to calculate the relative risks and the corresponding 95% confidence intervals with symptoms of depression as the main outcome variable. The models have been controlled for the exposure variable (ED consumption) and the covariates; perceived everyday pressure, school-related stress, general self-efficacy next to other background variables.

**Results:**

The sample comprised 63,233 (47.5%) boys and 66,621 (52.5%) girls. In total, 2.9% consumed ED daily, 52.3% had consumed any ED while 44.7% had never consumed ED. 18.3% of the total sample had a high level of symptoms of depression. Moreover, when adjusted for all variables, any (RR: 1.23, CI: 1.20–1.26) and daily ED consumption (RR: 1.94, CI: 1.85–2.03) were associated with increased symptoms of depression.

**Conclusion:**

ED continues to be a popular beverage among Norwegian adolescents and regular consumption of ED is related to key elements of adolescents’ symptoms of depression when adjusted by perceived everyday pressure, school-related stress and general self-efficacy. This study adds to the body of evidence linking regular ED consumption to mental health which is increasingly common among adolescents. Public health initiatives such as increasing public information or restricting ED sales to adolescents should be considered to reduce ED consumption among adolescents. Yet, further research is needed to understand the specific mechanisms of how ED and symptoms of depression are associated.

**Supplementary Information:**

The online version contains supplementary material available at 10.1186/s12889-025-23432-6.

## Introduction

Adolescence is an important period of life when it comes to influencing later food consumption and habits [1]. Energy drinks (ED) are effectively marketed as boosters of mental and physical health and have become a popular choice of beverage among adolescents with sales in Norway increasing by more than 20% annually [2–4]. ED are non-alcoholic beverages with an average caffeine content of 150 mg per litre, but can contain up to 320 mg per litre. Moreover, EDs often contain sugar, vitamins, minerals and amino acids in varying amounts [5, 6]. In Norway, ED labelling should contain a statement that these beverages are not recommended for children if they contain more than 150 mg of caffeine per litre. In addition, the caffeine content should be stated explicitly on the can [7]. Currently there are no government-imposed restrictions regarding who can purchase ED in Norway, though there have been made suggestions for this in recent time [8] where an agelimit has been sugested.

ED sales and consumption has increased among adolescents in Norway in recent years. According to Abel et al., 43% of the adolescents in lower and upper secondary schools consume EDs on a weekly basis [9]. They also found that adolescents usullay choose to consume the largest can (500 ml) when consuming ED which contains 160 mg of caffein [9]. This means that an adolescent with a bodyweight of 53 kg quickly exceeds the caffein dose of no concern set by the European Food Safety Authority (EFSA) and is at risk of sleep disturbance [6]. Moreover, our previous research demonstrated that ED consumption is associated with an increased risk of poor sleep [10]. Furthermore, studies on the reasons for consuming EDs have shown that adolescents usually consume EDs because “their friends do it”, “because they need energy”, and “to stay awake to do school work” [9, 11, 12].

There is a growing concern regarding the increased ED consumption in the population, particularly in relation to the consumption of ED among adolescents. In addition to sleep disturbance, ED has been linked to several adverse physical health effects such as headache and heart palpitation [3, 13, 14]. These effects are in addition to the known clinical symptoms of excess caffeine consumption such as nausea, increased heartrate and blood pressure to mention some [15]. In addition, studies have suggested that frequent ED consumption is associated with several mental health problems such as depression, stress and anxiety [16, 17]. Finally, Khouja et al. [18] found that ED consumption might be a marker of low well-being among children, though the direction of the association remains unclear.

There are several definitions of mental health and there has been an increased focus on both mental health and well-being in recent years. According to Goodman et al. “Mental health is defined as the level of psychological functioning including emotional, behaviour, attention and peer problems” [19]. The WHO further defines mental health as “a state of well-being that enables individuals to cope with the normal stresses of life, realize their abilities, work productively and contribute to their community. It encompasses emotional psychological and social wellbeing affecting how we think, feel and act.” [20]. Well-being on the other hand has no formal definition but one of the most cited definition is the one by Michaelson et al. which states that “Well-being can be understood as how people feel and how they function both on a personal and social level, and how they evaluate their lives as a whole” [21]. From the definitions of both mental health and well-being one can understand that mental health includes emotional, psychological and social components while well-being in addition includes physical and economic resources. Mental health is a part or component of well-being.

Adolescence is a developmental period characterized by several biological, psychological, and social changes [22, 23]. During adolescence, young people are vulnerable and prone to stress [24]. School stress and academic pressure strongly influence well-being and satisfaction of life during adolescence and are of all environmental stress factors, those most often reported by adolescents [25]. Thus, school success is one of the developmental goals of adolescence, which has a strong positive influence on well-being and satisfaction of life [26]. The association between stress factors and well-being again depends on several determinants such as the amount of the stressors counterbalanced by the adolescents’ resources, and level of self-efficacy [27].

Stress levels and self-efficacy among adolescents tend to change significantly during the transition into adulthood [28]. Self-efficacy refers to a person’s self-belief in their ability to maintain established goals, their ability to master challenges and daily life in general [29]. Burger et al. suggest that high levels of self-efficacy might reduce stress. Adolescents with low levels of self-efficacy may have difficulties handling school stress [28]. In contrast, good general self-efficacy (GSE) can positively influence adolescents to make certain health decisions and to promote physical activity, and it is considered an important health-promoting factor [30]. Consequently, self-efficacy is regarded an important aspect when trying to prevent mental health problems in adolescents. In the latest years, studies have indicated that adolescents experience increasing stress and pressure to perform well in school [31].

An increase in depressive symptoms and ED consumption among adolescents have been documented recently [4, 31, 32]. Yet there is limited knowledge on the potential link between ED consumption and different aspects of mental health such as symptoms of depression. Adolescence is a vulnerable developmental period where the potential negative effects of ED consumption can have a greater impact compared to later in life. Therefore, the objective of the current analyses is to investigate the association between symptoms of depression and ED consumption adjusted for GSE, school-related stress, perceived everyday pressure and other background variables in Norwegian adolescents between 2017 and 2022.

## Methods

### Study design and participants

For the present study, we used data from the Ungdata surveys 2017–2022. The Norwegian Ungdata (https://ungdata.no/english/) survey is an ongoing, national, annual, youth survey conducted at the municipal level. The survey is administered by the Norwegian Social Research (NOVA) at Oslo Metropolitan University in collaboration with the Regional Drug and Alcohol Competence Centres (KoRus)( https://korus.no/). Ungdata aims to document adolescent health and well-being and is typically performed every third year in each of the Norwegian municipalities. Participating adolescents are between 13 and 19 years of age (school grade 8–13). Information about ethical approval can be found in the declaration section at the end of this manuscript. The online survey is usually conducted within the scheduled school time in March and April each year. A teacher or public health nurse is present in the room while the students are completing their questionnaires. Due to the COVID-19 pandemic, data collection was interrupted from the 12th of March 2020 because of the national confinement and closure of schools. This resulted in a reduced number of participants in the year 2020. In 2021, the planned data collection resumed. The questionnaire comprises a section with mandatory variables used nationally in all participating municipalities, whereas other parts may be selected or de-selected by each municipality. Thus, the number of responses varies between mandatory and elective parts of the questionnaire. In the present study, the variables ED use and GSE were derived from the mandatory and elective parts of the survey, respectively.

Between 2017 and 2022, 571,933 responses were collected in total. The number of answers per year are given in Table 1. For the purposes of analyses, only adolescents from municipalities that chose to include the relevant questions on ED consumption, GSE, school-related stress, perceived everyday pressure, and depressive symptoms were eligible for the analytical sample. Thus, a total of 133,165 (133,165/571,933: 23.3%) answers were included in the final analysis as the data was used independent of the year they were collected. It should be noted that these sub-questionnaires were only offered to a selection of adolescents based on the choice of the municipality. In other words, not all adolescents had the opportunity to answer these questions.

**Table 1:** Number of answers given in Ungdata per year

	2017	2018	2019	2020	2021	2022	Total
**Responses Ungdata**	107,004	70,956	118,545	25,959	139,841	109,628	**571**,**933**
**Total answers current analysis**	36,463	6,006	82,095	1,192	5,461	1,948	**133**,**165**

### Measures

#### Depressive symptoms

Ungdata applies a six-item scale derived from the Depressive Mood Inventory as a measure of depressive symptoms, which is based on the Hopkins Symptoms Checklist. The measure shows good internal consistency with Cronbach’s α of 0.88, both in our analyses and in other studies [33, 34]. Participants were asked the following questions: In the past week have you been affected by any of the following: (1) “felt that everything is a struggle;” (2) “had sleep problems;” (3) “felt unhappy, sad, or depressed;” (4) “felt hopelessness about the future;” (5) “felt stiff or tense;” (6) “worried too much about things”. Participants could answer on a scale from 1 to 4 where 1 = “not been affected at all” to 4 = “been affected a great deal”. From this, mean scores were computed and a cut-off was set at ≥ 3 dichotomizing the results into either 1 = high level of depressive symptoms vs. 0 = low level of depressive symptoms [35].

#### Energy drink consumption

ED consumption was assessed by the question “How often do you drink energy drinks?” with answer options ranging from 1 (“never”) to 7 (“several times a day”). Responses to this question were categorized into “never”, “any” and “daily” ED consumption. Any ED refers to those who reported to have ever consumed ED up to 1–5 times a week, and daily ED consumption refers to those consuming ED ≥ 6 times a week.

#### GSE score

Self-efficacy was measured using the 5-item version of the GSE Scale which has been designed for the general population aged 12 years and older [36]. The scale aims to predict the ability of the respondent to manage and cope with daily demands. The following questions are asked: (1) “I always manage to solve difficult problems if I try hard enough”, (2) “If someone is opposing me, I can find methods and ways of getting what I want”, (3) “I feel confident that I could deal with unexpected events in an effective manner”, (4) “I stay calm when I encounter difficulties because I have confidence in my ability to master situations”, (5) “If I am in a predicament, I usually find a way out”. The answer options ranging from 1 to 4, where 1 = “completely disagree” and 4 = “completely agree”. The score is calculated by summing the individual’s scores on the five items. The score ranges from 5 to 20, with higher scores indicating higher self-efficacy. For the current analysis, we categorized the variable into four categories based on the values of GSE scores which is in line with previous studies [36]. The largest category was used as a reference category based on the distribution of respondents. The scale shows good internal consistency with Cronbach α of 0.80 (lower secondary school) and 0.88 (upper secondary school) dependent on the school grade level in our analyses and other studies [30]. In addition, other studies have also indicated high measures of internal consistency between the items in the GSE through psychometric analyses [37, 38].

#### Perceived everyday pressure

Perceived everyday pressure was measured using the general question “Do you feel pressure in your everyday life” with the different areas of pressure presented next: (1) To look good or have a good body; (2) To do well at school; (3) To do well at sports; (4) To have many followers and likes on social media. Answer options ranged from 0 to 4 with 0 = “no pressure” to 4 = “very much pressure”. These were then calculated to give a combined score between 0 and 16, which was subsequently subdivided into “little pressure” (0–4 points), “some pressure” (5–8 points) and “much pressure” (≥ 9 points) which is commonly done in Ungdata [31, 39].

#### School-related stress

School-related stress was measured using the question “I get stressed by schoolwork” with answer options ranging from 1 (“never”) to 5 (“very often”). The answer options “often” and “very often” were combined for the purpose of the current analysis.

#### Background variables

All participants reported on gender, school grade, and perceived family economy. The perceived family economy was measured by asking “Has your family’s economic situation been good or bad during the past two years?”, with five response options ranging from “always good” to “always bad”. For the purposes of the current analysis, the two lower options were merged into one category. School grade was used as a proxy for age due to anonymity concerns. These variables were used as background variables in the adjusted statistical analyses and were selected based on previous knowledge [10, 32].

### Statistical analysis

STATA version 17.0 was used for all statistical analyses. Symptoms of depression score was used as main outcome variable [40]. We calculated the relative proportions or relative risks (RR) and the corresponding 95% confidence intervals (95% CI) using multivariable Poisson regression models. In these analyses, we used the sandwich estimation method to generate robust standard errors [41–43]. The models were adjusted for sex, school grade and perceived family economy. A stepwise approach was used when analysing the associations between symptoms of depression and the different exposure variables. In Table 3, model 0 shows the unadjusted RRs while model 1 shows the RRs adjusted for background variables grade, gender and perceived family economy and Model 2 shows the full adjusted model. We also explored the associations in Model 2 by sex. The degree of multicollinearity was assessed by calculating the variance inflation factor (VIF) between the independent variables. There was no indication of multicollinearity (VIF < 1.5) in the models. Missingness was handled by listwise deletion of observations.

## Results

There were 133,165 responses that included data on ED consumption, depressive symptoms, school-related stress, everyday pressure and GSE which we included in the analyses. The characteristics of the participants are presented in Table 2. The sample comprised 63,233 (47.5%) boys and 66,621 (52.5%) girls. In total, 2.9% consumed ED daily, 52.3% had consumed any ED while 44.7% had never consumed ED. The majority of ED consumers, both any and daily, were found in grade 11. More than 40% of the adolescents perceived their family economy to be good, independent of their ED consumption habit. Of the adolescents that consumed ED daily experienced 50% of them little everyday pressure, but at the same time more than 50% experienced school-related stress. This distribution was similar for those consuming any ED and those never consuming ED. Most of the adolescents, independent of ED consumption category, scored low to mid on the GSE scale, but while this percentage was above 60% for those never consuming ED or any ED consumption, this was 47.1% for those consuming ED daily. Here, a larger percentage was found in the lowest score group (22.2%) compared to 15.4% in those never consuming ED and 16.1% in the any ED consumption group. Almost 20% of the total sample had a high level of symptoms of depression. Of those never consuming ED, 16.3% reported high levels of symptoms of depression, while 19.1% of those consuming any ED and 34.6% of those consuming ED daily reported the same symptoms.

**Table 2:** Description of the study participants by ED consumption category

	Never ED (*n* = 58,676)	Any ED (*n* = 68,417)	Daily ED (*n* = 3,821)	Total (*n* = 133,165)	*P*-values
Grade					< 0.0005
8	12,461 (21.2)	9,369 (13.7)	385 (10.1)	22,215 (17.0)	
9	10,606 (18.1)	11,414 (16.7)	559 (14.6)	22,579 (17.3)	
10	9,951 (17.0)	12,326 (18.0)	614 (16.1)	22,891 (17.5)	
11	10,376 (17.7)	14,614 (21.4)	940 (24.6)	25,930 (19.8)	
12	9,126 (15.5)	12,709 (18.6)	867 (22.7)	22,702 (17.3)	
13	6,156 (10.5)	7,985 (11.7)	456 (11.9)	14,597 (11.1)	
Perceived family economy					< 0.0005
Good	29,274 (49.8)	30,828 (44.7)	1,660 (43.3)	61,762 (46.9)	
Mostly good	18,406 (31.3)	22,464 (32.6)	1,067 (27.8)	41,937 (31.9)	
Neither/medium	8,691 (14.8)	11,926 (17.3)	701 (18.3)	21,318 (16.2)	
Mostly bad/bad	2,461 (4.2)	3,709 (5.4)	407 (10.6)	6,577 (5.0)	
Gender					< 0.0005
Boys	20,765 (35.7)	39,871 (58.7)	2,597 (68.8)	63,233 (48.7)	
Girls	37,404 (64.3)	28,038 (41.3)	1,179 (31.2)	66,621 (51.3)	
Perceived everyday pressure					< 0.0005
Little pressure	28,274 (47.5)	33,828 (48.5)	1,944 (50.0)	64,046 (48.1)	
Some pressure	18,479 (31.0)	21,356 (30.6)	974 (25.1)	40,809 (30.6)	
High pressure	12,829 (21.5)	14,514 (20.8)	967 (24.9)	28,310 (21.3)	
School-related stress					< 0.0005
Never	3,075 (5.2)	4,626 (6.6)	491 (12.6)	8,192 (6.1)	
Seldom	9,052 (15.2)	11,215 (16.1)	550 (14.2)	20,817 (15.6)	
Sometimes	18,325 (30.8)	21,205 (30.4)	873 (22.5)	40,403 (30.3)	
Often	29,130 (48.9)	32,652 (46.9)	1,971 (50.7)	63,753 (47.9)	
Depressive symptoms					< 0.0005
Low level	49,854 (83.7)	56,362 (80.9)	2,541 (65.4)	108,757 (81.7)	
High level	9,728 (16.3)	13,336 (19.1)	1,344 (34.6)	24,408 (18.3)	
GSE score					< 0.0005
Highest score	934 (1.6)	1,310 (1.9)	329 (8.5)	2,572 (1.9)	
Mid to high	12,228 (20.5)	14,089 (20.2)	865 (22.3)	27,182 (20.4)	
Low to mid	37,222 (62.5)	43,043 (61.8)	1,830 (47.1)	82,095 (61.7)	
Lowest score	9,198 (15.4)	11,256 (16.1)	861 (22.2)	21,315 (16.0)	

In the crude model, we found that both any and daily ED consumption were associated with increased RR of symptoms of depression with a RR of 1.17 (1.14, 1.20) and 2.12 (2.02, 2.22) respectively. After adjusting for sex, grade and family economy, the RR increased with any ED being 1.35 (1.32, 1.39) and daily ED being 2.51 (2.39, 2.63). While in Model 2, where we adjusted additionally for GSE, everyday pressure and school-related stress, we found that any and daily ED consumption was associated with increased RR of symptoms of depression with a RR of 1.23 (1.20, 1.26) and 1.94 (1.85, 2.03), respectively in the fully adjusted model. In addition, ED consumption was associated with a higher level of symptoms of depression, particularly among individuals with lower GSE scores. Those with the highest GSE scores had a significantly lower risk of depression symptoms (RR: 0.57, CI: 0.54, 0.59). The sex stratified analysis revealed similar associations as those described in Table 3 (appendix).

**Table 3:** Association between symptoms of depression and ED consumption adjusted for GSE, school-related stress, perceived everyday pressure and sociodemographic

Independent variables	Model 0 (RR (C)))	Model 1 (RR (CI))	Model 2 (RR (CI))
Energy drink consumption			
Never	Ref		
Any	1.17 (1.14, 1.20)	1.35 (1.32, 1.39)	1.23 (1.20, 1.26)
Daily	2.12 (2.02, 2.22)	2.51 (2.39, 2.63)	1.94 (1.85, 2.03)
Sociodemographic			
Family economy			
Good	Ref		
Mostly good	1.43 (1.39, 1.47)	1.33 (1.29, 1.37)	1.21 (1.17, 1.24)
Neither/medium	2.15 (2.08, 2.21)	1.85 (1.79, 1.90)	1.51 (1.47, 1.55)
Mostly bad/bad	3.44 (3.32, 3.56)	2.82 (2.72, 2.92)	1.94 (1.88, 2.01)
Grade			
8	Ref		
9	1.45 (1.38, 1.52)	1.36 (1.30, 1.43)	1.11 (1.06, 1.16)
10	1.81 (1.73, 1.90)	1.65 (1.58, 1.73)	1.27 (1.22, 1.32)
11	1.83 (1.75, 1.92)	1.62 (1.55, 1.69)	1.43 (1.38, 1.49)
12	1.93 (1.84, 2.02)	1.69 (1.62, 1.77)	1.50 (1.44, 1.56)
13	2.20 (2.10, 2.31)	1.84 (1.76, 1.93)	1.54 (1.48, 1.60)
Gender			
Male	Ref		
Female	2.79 (2.71, 2.87)	2.89 (2.82, 2.98)	1.40 (1.37, 1.44)
Psychological resource			
GSE score^1^			
Highest score	3.24 (3.10, 3.39)		2.19 (2.10, 2.29)
Mid to high	Ref		Ref
Low to mid	2.66 (2.60, 2.72)		1.78 (1.75, 1.83)
Lowest score	0.57 (0.54, 0.59)		0.79 (0.75, 0.83)
School-related stress			
Never	Ref		
Seldom	0.88 (0.78, 0.99)		0.80 (0.71, 0.89)
Sometimes	1.55 (1.40, 1.72)		1.07 (0.97, 1.19)
Often	6.79 (6.15, 7.49)		2.78 (2.52, 3.08)
Perceived everyday pressure			
Little	Ref		
Some	2.80 (2.71, 2.90)		1.78 (1.72, 1.85)
High	6.31 (6.11, 6.51)		2.92 (2.82, 3.08)

^1^Highest score; 19–20 points, Mid to high; 14–18 points, Low to mid; 8–13 points, Lowest score; 5–7 points. RR = relative risk. Model 0 shows the unadjusted values, model 1 shows adjusted for background variables, model 2 shows adjusted for all variables

## Discussion

Our main findings were, that adolescents who consumed ED had a higher likelihood of experiencing high level of depressive symptoms. This association was particularly evident in those reporting elevated levels of school-related stress and perceived everyday pressure. We found a non-linear association between GSE and depressive symptoms among ED consumers. While lower GSE scores were associated with higher risks of depressive symptoms, the highest GSE category demonstrated an increased risk compared to the middle category, highlighting the nuanced nature of this relationship. To our knowledge, this is the first study to report on symptoms of depression and ED consumption among adolescents in Norway.

We found that both any and daily ED consumption were positively associated with scores of depressive symptoms which is in line with previous research [14]. Daily ED consumption might contribute to a higher level of depressive symptoms or vice versa, a higher level of depressive symptoms may lead to adolescents consuming more ED. However, the direction of the association cannot be determined. In addition, we know from previous studies that ED use has been associated with poor sleep [44], which again might lead to poor mental and somatic health in adolescents [45, 46]. In the current study, we did not investigate this potential mediating effect between ED, sleep and symptoms of depression, as this was beyond the scope of the current analyses.

Some studies [18, 47] have reported that ED consumption might be a marker of low well-being. Other studies have indicated the importance of coping through self-efficacy to decrease the degree of depression and anxiety [48, 49]. Yet, our results indicate that those who reported a high GSE score were associated with having symptoms of depression which is in contrast with previous results from Norway [50]. However, we observed that the association from the crude regression analysis is supported even after accounting for potential confounding variables. This indicates that the relationship between ED consumption and the symptoms of depression is robust and not altered by the inclusion of other factors, like demographics, GSE, school-related stress, and perceived everyday pressure.

In recent years, there has been a steady increase in ED consumption among adolescents in Norway according to previous results [32]. In the current study, the overall ED consumption rate was high, particularly among boys which is in line with previous findings [14]. Most likely, there are several reasons for adolescents choosing to drink ED. These popular beverages are marketed as providing the ability to improve concentration and physical performance which can be a motivation by itself [51]. Also, the study by Trapp et al. [47] found that the home environment, peers and parents were important sources for influencing adolescents to consume ED overall. Moreover, social circumstances and interactions next to parental attitudes towards ED consumption have been shown to be important reasons for or situations in which ED are consumed [3].

Today’s adolescents are part of a generation, that experience pressure to perform and do well in different areas such as school, sports and social media [31]. Our results showed that increased perceived everyday pressure and school-related stress was associated with having symptoms of depression. At the same time, lower degree of school-related stress was not related to having symptoms of depression. School-related stress and perceived everyday pressure are variables which measure aspects of adolescents’ life that are most likely closely interrelated, and the effect measure might therefore go towards zero when both are included in regression analyses.

### Strengths and limitations

A major strength of the study is the large sample size with data from adolescents living all over Norway. However, there are some limitations to the study. We are unable to conclude on the direction of the association found as the data are observational and cross-sectional. In addition, the study might be subject to recall bias as the participants had to recall what took place in the past and thereby depending on the adolescent’s judgement and memory. Furthermore, the use of many different scales (the last 7 days, the last year or the last 2 years) contributes to the potential recall bias. However, we consider the risk of bias in relation to this as low. There may also be some bias in the material as only those who lived in the municipality that chose the optional part of the questionnaire had the opportunity to answer these questions. No adjustment was made for lack of independence between these observations. Finally, we only used two validated scales from the questionnaire, but the others are well established within Ungdata and are commonly used in this manner.

## Conclusion

In conclusion, results from our study show that attention should be paid to adolescents who consume ED on a regular basis as it is associated with having symptoms of depression. Education on possible risks of ED consumption could be part of health promotion and prevention measures directed to adolescents. In addition, restriction on sales of ED could be considered to reduce ED consumption among adolescents. Moreover, future research should investigate the potential short and long-term effects of regular ED consumption, since our data point towards an association between ED consumption and poor mental health. Especially, because adolescence is an important developmental period.

## Electronic supplementary material

Below is the link to the electronic supplementary material.

Supplementary Material 1

## Data Availability

Data may be obtained from a third party and are not publicly available. The data supporting our study are available from the Norwegian Centre for Research Data (NSD) and were used under license for the current study. The Ungdata survey is funded by The Norwegian Directorate of Health. Anonymous data have been made available for the authors by NOVA through NSD—the Norwegian Centre for Research Data.

## Abbreviations

CI: Confidence interval
ED: Energy drink
EFSA: European Food Safety Authority
GSE: General Self-Efficacy
KoRUS: Kompetansesenter for rusfeltet (Norwegian Competence Centre for Alcohol and Drug Abuse)
NOVA: Velferdsforskningsinstituttet (Norwegian Social Research)
NSD: Norwegian Centre for Research Data
RR: Relative risk
VIF: Variance inflation factor
