# Supplementary material for: Mental health and energy drink consumption among Norwegian adolescents; a cross-sectional study
Source: BMC Public Health. 2025 Jun 10;25:2158. doi: 10.1186/s12889-025-23432-6 (PMC12150526; doi:10.1186/s12889-025-23432-6)
Supplement: Supplementary file 1 — Supplementary Material 1 [file 12889_2025_23432_MOESM1_ESM.docx]

**Table 3a:** Model 2 stratified for boys: Association between symptoms of depression and ED consumption adjusted for GSE, school-related stress, perceived everyday pressure and sociodemographic.

| Independent variables | Model 2 |
| --- | --- |
| Energy drink consumption |  |
| Never | Ref |
| Any | 1.16 (1.10, 1.22) |
| Daily | 2.02 (1.86, 2.20) |
| Sociodemographic |  |
| Family economy |  |
| Good | Ref |
| Mostly good | 1.23 (1.16, 1.30) |
| Neither/medium | 1.61 (1.52, 1.72) |
| Mostly bad/bad | 2.29 (2.13, 2.46) |
| Grade |  |
| 8 | Ref |
| 9 | 1.12 (1.01, 1.23) |
| 10 | 1.38 (1.26, 1.52) |
| 11 | 1.72 (1.57, 1.88) |
| 12 | 1.91 (1.75, 2.08) |
| 13 | 1.94 (1.76, 2.13) |
| Psychological resource |  |
| GSE score^1^ |  |
| Highest score | 2.23 (2.03, 2.46) |
| Mid to high | Ref |
| Low to mid | 2.14 (2.03, 2.24) |
| Lowest score | 0.77 (0.71, 0.83) |
| School-related stress |  |
| Never | Ref |
| Seldom | 0.82 (0.70, 0.95) |
| Sometimes | 1.11 (0.97, 1.27) |
| Often | 3.17 (2.95, 3.61) |
| Perceived everyday pressure |  |
| Little | Ref |
| Some | 1.86 (1.76, 1.97) |
| High | 3.13 (2.94, 3.33) |

**Table 3b:** Model 2 stratified for girls: Association between symptoms of depression and ED consumption adjusted for GSE, school-related stress, perceived everyday pressure and sociodemographic

| Independent variables | Model 2 |
| --- | --- |
| Energy drink consumption |  |
| Never | Ref |
| Any | 1.26 (1.23, 1.29) |
| Daily | 1.80 (1.70, 1.90) |
| Sociodemographic |  |
| Family economy |  |
| Good | Ref |
| Mostly good | 1.19 (1.16, 1.23) |
| Neither/medium | 1.47 (1.42, 1.51) |
| Mostly bad/bad | 1.80 (1.74, 1.87) |
| Grade |  |
| 8 | Ref |
| 9 | 1.11 (1.06, 1.16) |
| 10 | 1.24 (1.19, 1.30) |
| 11 | 1.36 (1.30, 1.42) |
| 12 | 1.39 (1.34, 1.46) |
| 13 | 1.44 (1.37, 1.51) |
| Psychological resource |  |
| GSE score^1^ |  |
| Highest score | 2.18 (2.08, 2.28) |
| Mid to high | Ref |
| Low to mid | 1.70 (1.66, 1.74) |
| Lowest score | 0.83 (0.78, 0.88) |
| School-related stress |  |
| Never | Ref |
| Seldom | 0.67 (0.56, 0.80) |
| Sometimes | 0.82 (0.70, 0.96) |
| Often | 2.03 (1.74, 2.36) |
| Perceived everyday pressure |  |
| Little | Ref |
| Some | 1.70 (1.62, 1.78) |
| High | 2.78 (2.67, 2.90) |
